# Inhibition of mammalian target of rapamycin complex 1 in the brain microvascular endothelium ameliorates diabetic Aβ brain deposition and cognitive impairment via the sterol‐regulatory element‐binding protein 1/lipoprotein receptor‐associated protein 1 signaling pathway

**DOI:** 10.1111/cns.14133

**Published:** 2023-03-08

**Authors:** Gege Jiang, Zhenzhen Long, Yaoling Wang, Yaofeng Wang, Ping Xue, Minfang Chen, Kang Yang, Wei Li

**Affiliations:** ^1^ Department of Geriatrics, Union Hospital, Tongji Medical College Huazhong University of Science and Technology Wuhan China; ^2^ Xiang Yang No. 1 People Hospital Affiliated Hospital of Hubei University Medicine XiangYang China; ^3^ Department of Hepatobiliary Surgery, Union Hospital, Tongji Medical College Huazhong University of Science and Technology Wuhan China; ^4^ Department of Geriatrics, Li‐Yuan Hospital, Tongji Medical College Huazhong University of Science and Technology Wuhan China

**Keywords:** amyloid‐β, brain microvascular endothelial cells, cognitive impairment, diabetes, mTORC1

## Abstract

**Aims:**

Mammalian target of rapamycin complex 1 (mTORC1) is highly activated in diabetes, and the decrease of low‐density lipoprotein receptor‐associated protein 1 (LRP1) in brain microvascular endothelial cells (BMECs) is a key factor leading to amyloid‐β (Aβ) deposition in the brain and diabetic cognitive impairment, but the relationship between them is still unknown.

**Methods:**

In vitro, BMECs were cultured with high glucose, and the activation of mTORC1 and sterol‐regulatory element‐binding protein 1 (SREBP1) was observed. mTORC1 was inhibited by rapamycin and small interfering RNA (siRNA) in BMECs. Betulin and siRNA inhibited SREBP1, observed the mechanism of mTORC1‐mediated effects on Aβ efflux in BMECs through LRP1 under high‐glucose conditions. Constructed cerebrovascular endothelial cell‐specific Raptor‐knockout (Raptor^fl/+^) mice to investigate the role of mTORC1 in regulating LRP1‐mediated Aβ efflux and diabetic cognitive impairment at the tissue level.

**Results:**

mTORC1 activation was observed in HBMECs cultured in high glucose, and this change was confirmed in diabetic mice. Inhibiting mTORC1 corrected the reduction in Aβ efflux under high‐glucose stimulation. In addition, high glucose activated the expression of SREBP1, and inhibiting of mTORC1 reduced the activation and expression of SREBP1. After inhibiting the activity of SREBP1, the presentation of LRP1 was improved, and the decrease of Aβ efflux mediated by high glucose was corrected. Raptor^fl/+^ diabetic mice had significantly inhibited activation of mTORC1 and SREBP1, increased LRP1 expression, increased Aβ efflux, and improved cognitive impairment.

**Conclusion:**

Inhibiting mTORC1 in the brain microvascular endothelium ameliorates diabetic Aβ brain deposition and cognitive impairment via the SREBP1/LRP1 signaling pathway, suggesting that mTORC1 may be a potential target for the treatment of diabetic cognitive impairment.

## Graphical abstract

Raptor^fl/+^ mice are used to exclude the effects of other brain cells and tissues and to avoid the manipulation of mTORC2 signaling. The results first confirm that Mammalian target of rapamycin complex 1 in the brain microvascular endothelial ameliorates diabetic Aβ brain deposition and cognitive impairment via SREBP1/LRP1 signal pathway.

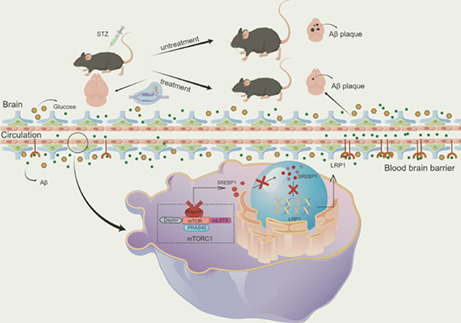

## 1 INTRODUCTION

Cognitive impairment, which is one of the most common chronic complications of diabetes, severely damages patient health. Epidemiological studies have shown that diabetes is associated with dementia^1^ and mild cognitive impairment.^2^ A follow‐up study showed that diabetes mellitus can accelerate the decline of cognitive function.^3^ The incidence of dementia caused by mild cognitive impairment in diabetic patients was 8.43%,^4^ and cognitive impairment in elderly diabetic patients was as high as 31.5%.^5^ A large number of evidence‐based medicine studies showed that diabetes is a powerful and independent risk factor for cognitive impairment and late‐onset Alzheimer's disease (AD).^1^, ^6^, ^7^, ^8^ Diabetic cognitive impairment is characterized by acquired cognitive and behavioral defects, which are mainly manifest as decreased learning ability, memory impairment, and slow response,^1^, ^6^ and are aggravated with age.

There are characteristic pathological changes associated with AD in the brain tissue of patients with diabetic cognitive impairment, such as amyloid plaques (mainly amyloid‐β), neuronal fibers, and entanglement.^9^ Many studies have shown that diabetes causes learning and memory impairment^10^ by increasing amyloid‐β (Aβ) deposition in brain tissue. Furthermore, reducing Aβ deposition with drugs could significantly improve the cognitive function of diabetic mice.^11^ The normal brain has multiple mechanisms for Aβ clearance, such as degradation clearance, blood–brain barrier clearance, interstitial fluid bulk‐flow clearance, and cerebrospinal fluid absorption clearance. The clearance mechanisms cause different degrees of damage with aging, the dynamic balance of Aβ levels in the brain is maintained in the early stage of life.^12^ A great number of studies have shown that nearly 50% of Aβ in the brain is transported to the peripheral blood through the blood–brain barrier,^13^ which has been confirmed in animal models.^14^, ^15^ Studies have shown that endothelial dysfunction is an important cause of blood–brain barrier damage and Aβ deposition in the brain, and the downregulation of low‐density lipoprotein receptor‐related protein 1 (LRP1) in brain endothelial cells plays an important role in Aβ clearance and promotes diabetic cognitive impairment.^16^ In addition, LRP1 levels decrease with age and AD and are closely related to Aβ deposition in the brain, suggesting that maintaining or increasing LRP1 levels during diabetes may be a potential strategy for treating and preventing diabetic cognitive dysfunction. This information prompted us to identify the specific mechanism by which LRP1‐mediated Aβ efflux is regulated in diabetes.

As a multisubunit complex of mammalian target of rapamycin (mTOR), mammalian target of rapamycin complex 1 (mTORC1) is composed of regulatory associated protein of mTOR (Raptor), proline‐rich AKT substrate 40 kDa (PRAS40), and mammalian lethal with sec‐13 (mLST8). Raptor is an mTOR‐binding protein that regulates mTORC1 activity by binding to the downstream mTORC1 substrates phosphoprotein 70 ribosomal protein S6 kinase (P70S6K) and 4 E binding protein 1 (4EBP1) and promotes translation and protein synthesis.^17^ It has been demonstrated that the mTORC1 signaling pathway plays an important role in memory, the maintenance of synaptic plasticity^18^ and neurodegenerative diseases.^19^ mTORC1 activation is an early biochemical change in the hippocampus of AD patients, and its activation level is consistent with the degree of cognitive impairment in patients.^20^ The mTORC1 signaling pathway plays an important role in regulating autophagy and Aβ deposition in the brain during diabetes.^21^ However, how mTORC1 regulates LRP1 and ultimately leads to Aβ translocation through the blood–brain barrier and diabetic cognitive impairment remains unclear. Herein, we elucidated the mechanism by which inhibiting mTORC1 in the brain microvascular endothelium ameliorates diabetic Aβ brain deposition and cognitive impairment via the SREBP1/LRP1 signaling pathway.

## 2 MATERIALS AND METHODS

### 2.1 Animals

We crossed Raptor^tm1.1Dmsa/J^ mice with SP‐A Cre transgenic mice that express Cre recombinase in cerebrovascular endothelial cells to generate cerebrovascular endothelial cell‐specific knockout mice (Raptor^fl/fl^). Due to the Raptor^fl/fl^ genotype, Cre + mice did not develop after birth, and so they could not eat and died. Therefore, we used Raptor^fl/+^; Cre+ (Raptor^fl/+^) male mice as the experimental group and wild‐type male mice as the control group. The experimental mice were housed in a specific pathogen‐free environment with free access to food and light and dark cycles for 12 h. After 1 week of adaptation, wild‐type mice and Raptor^fl/+^ mice were randomly divided into the control and diabetic groups. Diabetic mice were intraperitoneally injected with 60 mg/kg/d STZ for five consecutive days, and the control group were injected with the same dose of sodium citrate buffer. After 1 week, mice with blood glucose ≥16.7 mmol/L were considered as having type 1 diabetes and were prepared for subsequent experiments. There were four groups of animals, including the control group, Raptor^fl/+^ group, diabetes group, and Raptor^fl/+^ diabetes group, with 15 mice in each group. Finally, excluding accidental death and modeling failure, the number of mice in each group was 8, 12, 9, and 8, respectively. This study conformed to the principles of animal protection and institutional ethics in China. All animal protocols were approved by the Animal Care and Use Committee, Tongji Medical College, Huazhong University of Science and Technology. The IACUC approval number was [2019] 2570.

### 2.2 Reagents and antibodies

Details can be found in Supplementary Material S1.

### 2.3 Cell culture and treatment

Human brain microvascular endothelial cells (HBMECs) were purchased from ScienCell Research Laboratories (ScienCell 1000) and cultured in 1640 medium supplemented with 10% fetal bovine serum according at 37°C in a humidified atmosphere (5% CO_2_ and 95% air) to the recommendations. Cells were cultured in starvation solution for 24 h before various treatments. HBMECs were pretreated with rapamycin (20 nM) and Betulin (50 mM) for 1 h before high‐glucose treatment.

### 2.4 In vitro blood–brain barrier model

To create an in vitro blood–brain barrier model, HBMECs were seeded at a density of 5 × 10^4^/cm^2^ on top of semipermeable Transwell polycarbonate tissue culture inserts (pore size 0.4 μm and bottom diameter 6.5 mm) (Corning Costar Corp.). The fluid was changed every 2 days. The transendothelial resistance was monitored by an EVOM epithelial voltmeter with an STX2 electrode (World Precision Instruments). When the resistance value peaked, the cells formed a continuous monolayer and were used as a blood–brain barrier model for subsequent functional experiments (Figure S1).

### 2.5 Behavioral experiment

#### 2.5.1 Fear conditioning test

Training was performed 1 day before the test. The mice were given a 3‐min adaptation period in a Skinner chamber (Xinruan Information Technology Co., Ltd) to eliminate exploratory reflexes as follows: 20 s of noise stimulation, and at the end of the noise during the last 2 s and electric shock (0.5 mA) was administered at intervals of 30 s after repeated noise stimulation and electric shock, for a total of five cycles. After 24 h, the mice were placed in a Skinner box, and the percentage of time the mice spent freezing (freezing time) was recorded within 5 min.

#### 2.5.2 Novel object recognition test

The new object recognition experiment included an adaptation period, familiarity period, and test period, for a total of 3 days. The mice were placed in a test room 3 days in advance to eliminate the stress response. On Day 1, the mice were placed in the test box without any objects allowed to move freely for 5 min. On Day 2, after 30 min of adaptation, the mice were placed in a test box with two identical objects (A and B) in the two corners, and then the mice were placed back in the box with the two objects in the middle of the test box. Then the mouse's nose or mouth was ≤2 cm away from the object indicated observation, and the exploration of the two objects within 5 min was recorded by the Ethovision XT animal trajectory tracking system (Ranzhe Instrument Equipment Co., Ltd). On Day 3, object B was replaced with object C, which had a different shape, and the remaining steps were the same as those performed on Day 2. The exploration time of objects A and C was recorded as tA and tC, respectively. The test chamber was wiped with 75% alcohol before each mouse and object were placed to eliminate odor effects. The formula of to determine the cognitive index was RI = tC/(tA + tC) × 100%.

### 2.6 Tissue and brain microvessel preparation

After the behavioral experiment, three mice in each group were anesthetized. After successful anesthesia, the mice were perfused with 4°C saline until the liver, lung, and tongue were white and then were perfused with 4% paraformaldehyde in the original position until the mice were stiff. After the brain tissue was removed, it was immersed in 4% paraformaldehyde and fixed overnight at 4°C. Six mice in each group were immediately frozen in liquid nitrogen and stored at −80°C for Western blot, qRT‐PCR, and ELISA analysis. The experimental method to prepare brain microvessels was described previouely.^22^ The brain tissue was stored at −80°C and placed in a grinder to prepare powder, and the powder was dissolved in precooled PBS and centrifuged at 4°C and 500 *g* for 5 min. The obtained tissue was added to 18% dextran and centrifuged at 4°C and 2500 *g* for 20 min to obtain the initial sample of brain microvasculature. Then, the sample was resuspended in PBS and filtered through a 40 μm cell filter. The brain microvasculature at the top of the filter was used for the experiment.

### 2.7 Immunofluorescence

We performed immunofluorescence staining on brain tissue sections and HBMECs. The tissue sections were dewaxed with xylene and then rehydrated in ethanol. High glucose‐treated HBMECs were fixed on cell slides with 4% paraformaldehyde. The fixed sections and cell slides were transferred to blocking solution (goat serum) for 1 h. The blocked HBMECs were incubated overnight at 4°C with primary antibodies against SREBP1 (AF6283, diluted 1:100, Affinity Biosciences) and p‐mTOR (cst5536, diluted 1:100, Cell Signaling Technology). The tissue sections were incubated overnight at 4°C with primary antibodies against SREBP1 (AF6283, diluted 1:200, Affinity Biosciences), LRP1 (ab92544, diluted 1:500, Abcam), Raptor (DF7527, diluted 1:200, Affinity Biosciences) and CD31 (ab182981, diluted 1:2000, Abcam). Immunoreactions were observed using CY3‐conjugated affinity‐pure goat anti‐rabbit IgG (BA1032, diluted 1:100, Bost Biotech) and FITC‐conjugated affinity‐pure goat anti‐mouse IgG (BA1011, diluted 1:100, Bost Biotech). Nuclei were stained with Hoechst (Invitrogen). Antifluorescent quenchers were added, and the slides were observed under a microscope (Olympus CX31 microscope).

### 2.8 Immunohistochemistry

Tissue sections were dewaxed with xylene and gradiently hydrated in ethanol for antigen repair. Endogenous peroxidase was blocked with 3% hydrogen peroxide at room temperature for 20 min, and the sections were then incubated in 10% goat serum for 30 min. The washed sections were incubated with primary antibodies against Aβ (sc‐28365, diluted 1:10, Santa Cruz Biotechnology) at 4°C overnight. On the next day, the sections were reacted with HRP‐labeled goat anti‐Ig G polymer for 30 min, and DAB was used to observe the color reaction. Target detection was performed under a microscope (Olympus CX31 microscope).

### 2.9 Western Blot analysis

The lysis buffer (Boster Biological Technology) was added to the prepared tissue and cell samples and was lysed at 4°C for 30 min and nuclear and cytoplasmic protein extraction kits (Beyotime Biotechnology) were used to separate cytoplasmic and nuclear proteins. The lysate was collected and centrifuged at 13,902 *g* for 10 min. The supernatant protein concentration of the supernatant was determined by a BCA kit (Boster Biological Technology) to ensure the same quality of each sample. After being leveled, the supernatant was fully mixed with loading buffer and boiled for 10 min. Equal amounts of denatured supernatant were transferred to a nitrocellulose membrane after SDS‐PAGE, blocked with 5% skimmed milk powder, and incubated with the corresponding primary antibody and enzyme‐labeled secondary antibody. Protein expression was observed with an enhanced chemiluminescence detection reagent. The results were recorded by a ChemiDoc imaging system (Bio‐Rad) and analyzed quantitatively by ImageJ software.

### 2.10 Quantitative RT‐PCR

Total RNA was extracted from prepared cerebral microvessels using TRIzol reagent. cDNA was reverse transcribed using PrimeScript RT Master Mix. A SYBR Premix Ex Taq kit and an iCycler Real‐Time PCR detection system (Bio‐Rad) were used for quantitative PCR. RNA expression levels were analyzed by normalizing to β‐actin, and the value was determined by the 2‐ΔΔCt method. The primer sequences can be found in Table S1.

### 2.11 Amyloid‐β (1‐40/42) peptide transport studies

As described previously,^23^ we determined the transport process of Aβ from the basolateral to apical (B → A) side. Briefly, 0.1 nM TAMRA‐Aβ40/42 and 0.05 mM FITC‐inulin were added to the basolateral chamber to measure the transport rate of Aβ from the basolateral to apical side. After 6 h of incubation with HBMECs, media in both compartments were separately collected to measure the absorbance values of TAMRA‐Aβ40/42 and FITC‐inulin absorbance. The clearance quotient (CQ) of B → A (TAMRA‐Aβ40/42 CQB → A) transport was calculated using the following equations.

$$\text{TAMRA}-\mathrm{A\beta}40/42\mathrm{CQB}\to A=\left(\frac{\frac{\text{TRAMA}-\mathrm{A\beta}40/42\;\text{in apical chamber}}{\text{TRAMA}-\mathrm{A\beta}40/42\;\text{total}}}{\frac{\text{FITC}-\text{inulin in apical chamber}}{\text{FITC}-\text{inulin total}}}\right)$$

Total TRAMA‐Aβ40/42 and total inulin were the sums of the apical and basolateral chambers. TRAMA‐Aβ40/42: Abs/Em = 544/572 mm; FITC‐inulin: Abs/Em = 490/520 mm.

### 2.12 Amyloid‐β (1–40/42) peptide ELISA

Experimental procedures were performed based on the product instructions. Briefly, the standard sample was diluted to 50 μL, and the concentrations were 120, 80, 40, 20, and 10 pg/mL. The blank and sample wells were set up, and the sample was added to make a final 5× dilution of the sample. The enzyme was added, and the plate was incubated and washed, after which the color liquid was added and incubated at 37°C in the dark for 10 min, and the reaction was terminated. The absorbance (OD value) of each well was measured at a wavelength of 450 nm wavelength after zeroing with the blank well.

### 2.13 Statistical analyses

Statistical analyses were performed using GraphPad Prism 7.0 software. The Shapiro–Wilk test was used to verify the normality of the distribution of the data. Significant differences between the two groups were analyzed by unpaired *t*‐test, and data between multiple groups were analyzed by one‐way analysis of variance (ANOVA) and Tukey's post hoc test. Two‐way ANOVA and Bonferroni post hoc correction were used for multiple comparisons. The relevant experimental results were verified at least three times and are displayed as the mean ± SEM. If *p* < 0.05, the difference was considered significant. In addition, the graphical abstract was created with FigDraw.

## 3 RESULTS

### 3.1 High glucose activates mTORC1 in the cerebral vascular endothelium in vivo and in vitro

**FIGURE 1:**
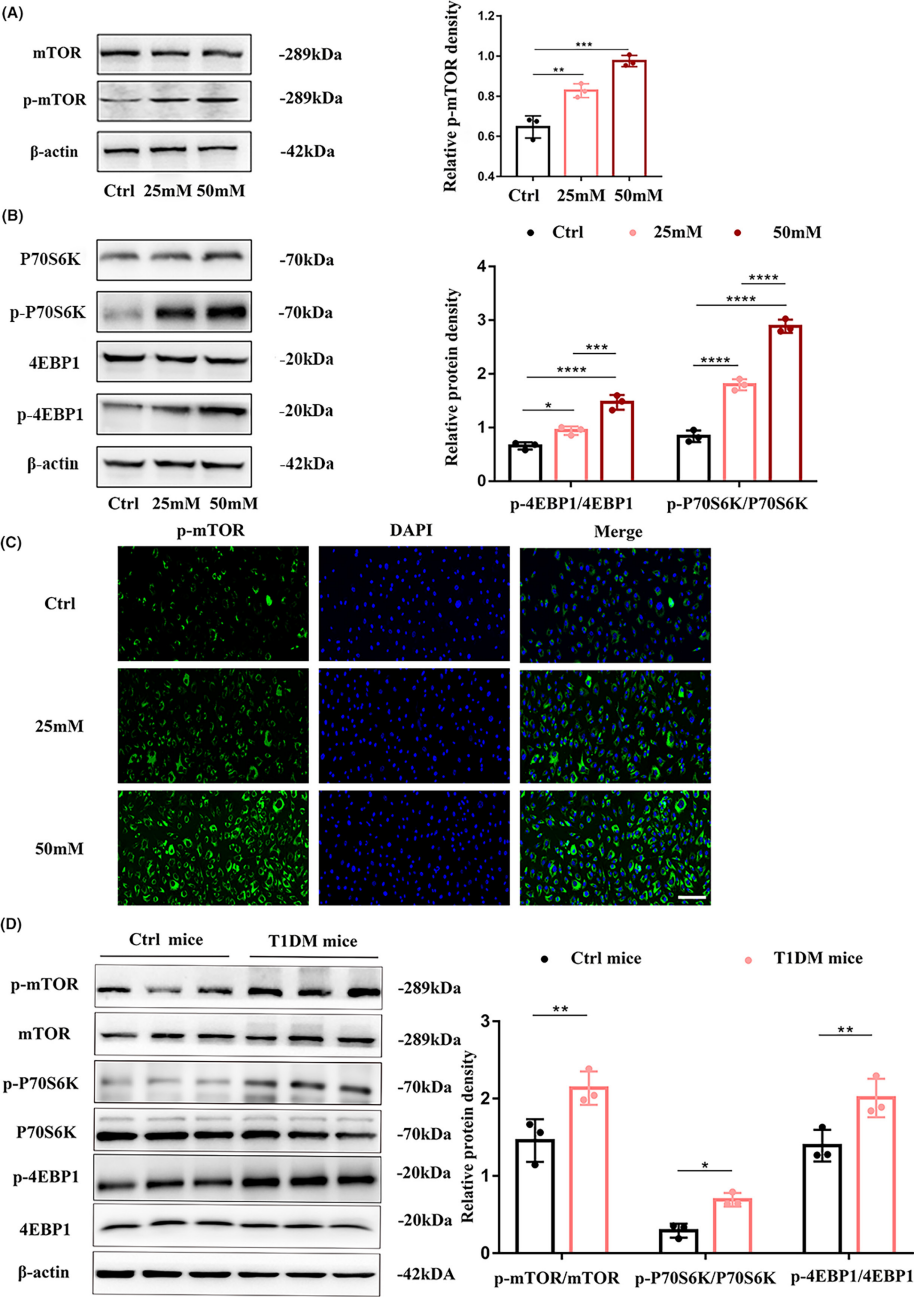
High glucose activates mTORC1 in the cerebral vascular endothelium in vivo and in vitro. Western blot analysis of (A) p‐mTOR (phosphorylated mTOR), mTOR, (B) p‐P70S6K (phosphorylated P70S6K), p‐4EBP1 (phosphorylated 4EBP1), P70S6K, and 4EBP1 in HBMECs treated with 25 and 50 mM glucose for 72 h (left). (A, B) The corresponding bar graph (right) shows the quantification of phosphorylated protein levels by the ratio of phosphorylated protein to total protein. (C) Immunofluorescence staining of p‐mTOR (green) in HBMECs stimulated with 25 and 50 mM glucose for 72 h. Cell nuclei were stained with Hoechst (blue). Scale bar: 100 μm. (D) p‐mTOR (phosphorylated mTOR), mTOR, p‐P70S6K (phosphorylated P70S6K), p‐4EBP1 (phosphorylated 4EBP1), and P70S6K, 4EBP1 protein levels in the brain microvasculature of diabetic mice were measured by Western blotting. The bar graph shows the relative expression presented as the ratio to β‐Actin. *n* = 3 mice per group. All data are repeated three independent experiments. *p < 0.05, **p < 0.01, ***p < 0.001 and ****p < 0.0001.

It is known that the activation of mTORC1 in the hippocampus occurs in the early stage of AD and is consistent with the degree of cognitive impairment in AD patients.^20^ As an important part of the blood–brain barrier, cerebrovascular endothelial cells play an important role in promoting cognitive impairment in diabetic patients.^16^ To investigate the relationship between mTORC1 and cerebrovascular endothelial cells in the diabetic state, we examined mTORC1 in vitro and in vivo. First, under high‐glucose stimulation, the expression of phospho‐mTOR increased significantly with increasing glucose concentrations, and the phosphorylation levels of the markers P70S6K and 4EBP1, which represent mTOR activation, also increased (Figure 1A–C). Then, in the brain microvasculature of diabetic mice, we observed outcomes that were consistent with the in vitro results (Figure 1D). These results showed that high glucose activated mTORC1 in the cerebral vascular epithelium in vivo and in vitro.

### 3.2 mTORC1 impairs Aβ efflux in HBMECs under high‐glucose conditions by downregulating LRP1 expression

**FIGURE 2:**
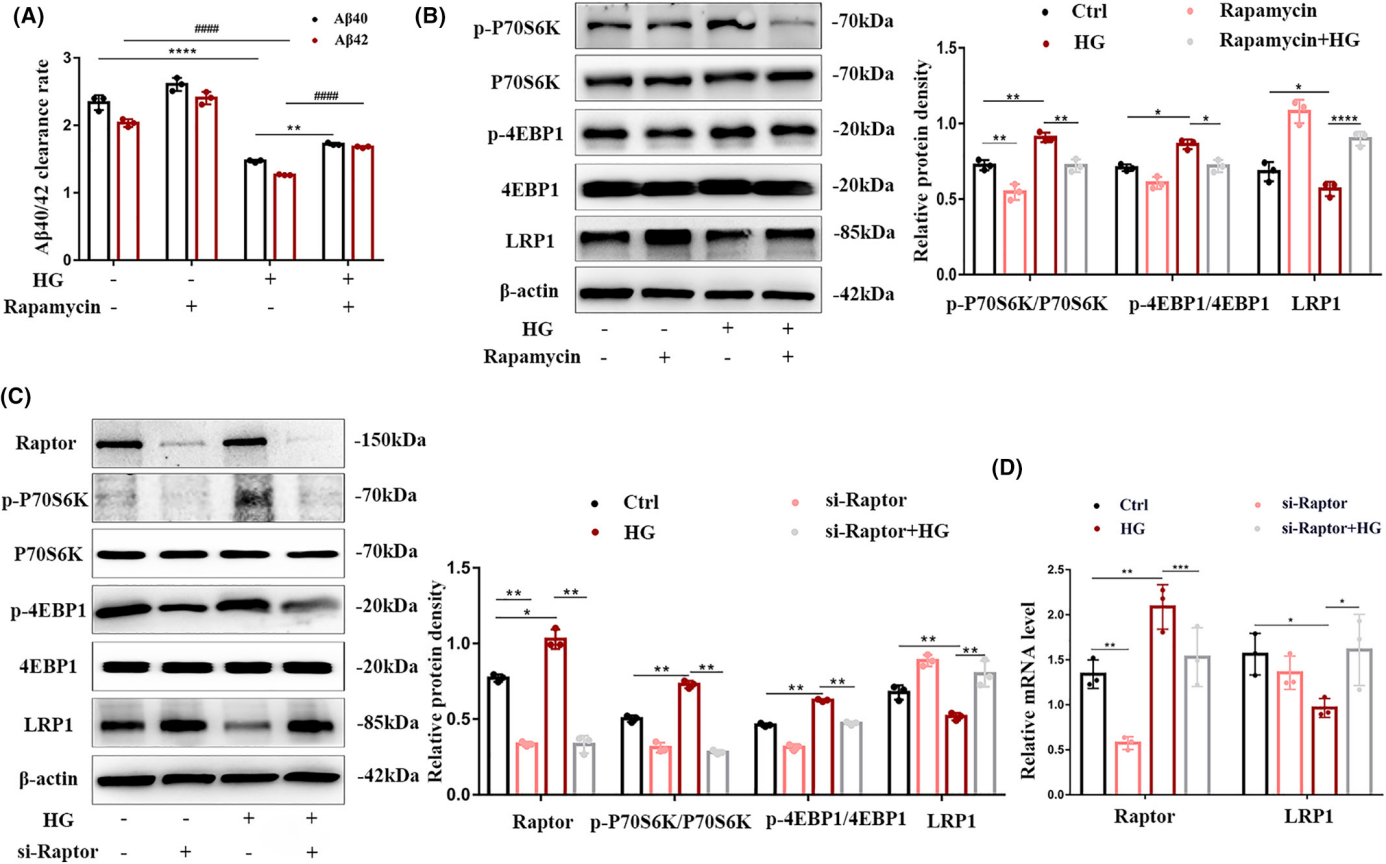
mTORC1 impairs Aβ efflux in HBMECs under high‐glucose conditions by downregulating LRP1 expression. Cultured HBMECs pretreated with or without rapamycin (20 nmol/L) were stimulated with 50 mM glucose. After 72 h, (A) the efflux rate of Aβ was evaluated in a Transwell in vitro blood–brain barrier model. (B) The protein levels of p‐P70S6K, P70S6K, p‐4EBP1, 4EBP1, and LRP1 in whole cell lysates were detected by Western blotting (left). The bar graph shows the quantification of all target proteins (right). HBMECs were transfected with Raptor siRNA (si‐Raptor) and the corresponding negative control (Ctrl.), followed by 50 mM glucose stimulation for 72 h, and then lysis. (C) Western blotting with Raptor, p‐P70S6K, P70S6K, p‐4EBP1, 4EBP1, and LRP1 antibodies was performed, and (D) relative mRNA expressions were detected by quantitative reverse transcription–polymerase chain reaction (qRT‐PCR). mRNA expression was normalized to β‐actin. All data were obtained from three independent experiments. *, ^#^ *p* < 0.05; **, ^##^ *p* < 0.01; ***, ^###^ *p* < 0.001 and ****; ^####^ *p* < 0.0001.

The role of cerebral vascular endothelial LRP1 in mediating Aβ deposition has been widely recognized. We observed the activation of mTORC1 in the diabetic cerebrovascular endothelium. To explore whether mTORC1 regulates LRP1‐mediated Aβ efflux, some experiments were performed. First, we used rapamycin to inhibit mTORC1 in HBMECs and observed that the efflux of Aβ40 and Aβ42 damaged by high glucose was improved by rapamycin (Figure 2A), and transport was from the basolateral to the apical side of the Transwell simulation model. Next, we used rapamycin and Raptor‐siRNA (si‐Raptor) to interfere with mTORC1. Western blotting (Figure 2B,C) and qRT‐PCR (Figure 2D) showed that after mTORC1 was inhibited, the downregulation of LRP1 expression induced by high glucose was reversed at the protein and gene levels. These results showed that mTORC1 damaged Aβ efflux from HBMECs under high‐glucose conditions by downregulating the expression of LRP1.

### 3.3 mTORC1 regulates the expression and activation of SREBP1 in HBMECs under high‐glucose conditions

**FIGURE 3:**
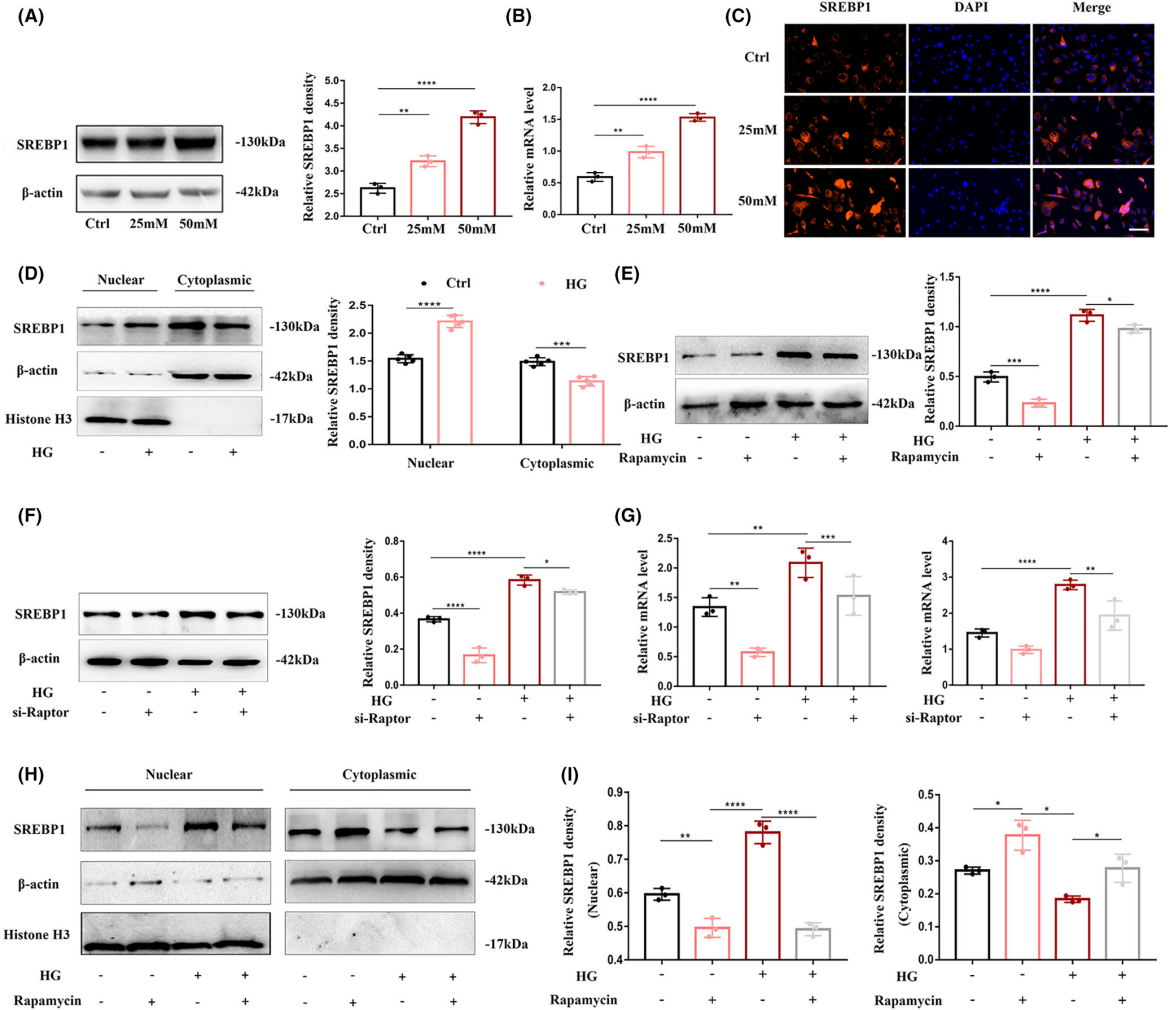
mTORC1 regulates the expression and activation of SREBP1 in HBMECs under high‐glucose conditions. (A) Western blot analysis of SREBP1 in HBMECs stimulated with 25 and 50 mM glucose for 72 h (left). β‐Actin was used as a loading control. The bar graph (right) shows the densitometric analysis. (B) After HBMECs were cultured in 25 and 50 mM glucose for 72 h, SREBP1 was detected by quantitative reverse transcription–polymerase chain reaction (qRT‐PCR). mRNA expression was normalized to β‐actin. (C) Immunofluorescence staining of SREBP1 (red) in HBMECs stimulated with 25 and 50 mM glucose for 72 h. Cell nuclei were stained with Hoechst (blue). Scale bar: 50 μm. (D) Cytoplasmic and nuclear extracts from HBMECs treated with 50 mM glucose were immunoblotted using anti‐SREBP1 (left). Bar graphs show densitometric analysis of SREBP1 expression in cytoplasmic extracts and nuclear extracts (right). Cultured HBMECs pretreated with or without rapamycin (20 nmol/L) were stimulated with 50 mM glucose. (E) After 72 h, the protein levels of SREBP1 in the whole cell lysate were detected by Western blotting (left). The bar graph shows the quantification of SREBP1 (right). HBMECs were transfected with Raptor siRNA (si‐Raptor) and the corresponding negative control (Ctrl.), followed by 50 mM glucose stimulation for 72 h, and then lysis. (F) Western blotting with SREBP1 antibody and (G) relative Raptor (left) and SREBP1 (right) mRNA expressions were detected by quantitative reverse transcription–polymerase chain reaction (qRT‐PCR). mRNA expression was normalized to β‐actin. (H) Cultured HBMECs pretreated with or without rapamycin (20 nmol/L) were stimulated with 50 mM glucose, and cytoplasmic and nuclear extracts were immunoblotted using anti‐SREBP1. (I) Bar graphs show the densitometric analysis of SREBP1 expression in cytoplasmic extracts and nuclear extracts. Data are shown from at least three independent experiments. *p < 0.05; **p < 0.01; ***p < 0.001 and ****p < 0.0001.

Studies have shown that in diabetes, mTORC1 can be activated to induce the transcriptional expression and functional activation of Sterol‐regulatory element binding protein 1 (SREBP1).^24^ Therefore, we hypothesized that mTORC1 can also regulate SREBP1 in cerebrovascular endothelial cells. To confirm this hypothesis, we conducted experiments, and our results showed that under high‐glucose stimulation, the levels of SREBP1 increased significantly in a concentration‐dependent manner (Figure 3A,B). In addition, we performed Western blot analysis of the cytoplasm and nucleoproteins, and found that SREBP1 translocated from the cytoplasm to the nucleus under high‐glucose stimulation (Figure 3D), indicating that high glucose could activate SREBP1 transcription and activation, which was also confirmed by immunofluorescence staining (Figure 3C). After confirming the activation of SREBP1 by high glucose, we used rapamycin and Raptor gene silencing to inhibit the activity of mTORC1 to explore its relationship with SREBP1. After inhibiting mTORC1 activity, the upregulation of SREBP1 caused by high glucose was partially corrected (Figure 3E–G). Interestingly, analysis of cytoplasm and nuclear protein levels in cells treated with rapamycin showed that inhibiting mTORC1 activation significantly reduced the nuclear accumulation of SREBP1 induced by high glucose in HBMECs (Figure 3H,I).

### 3.4 SREBP1 damages the Aβ efflux of HBMECs under high glucose conditions by downregulating LRP1 expression

**FIGURE 4:**
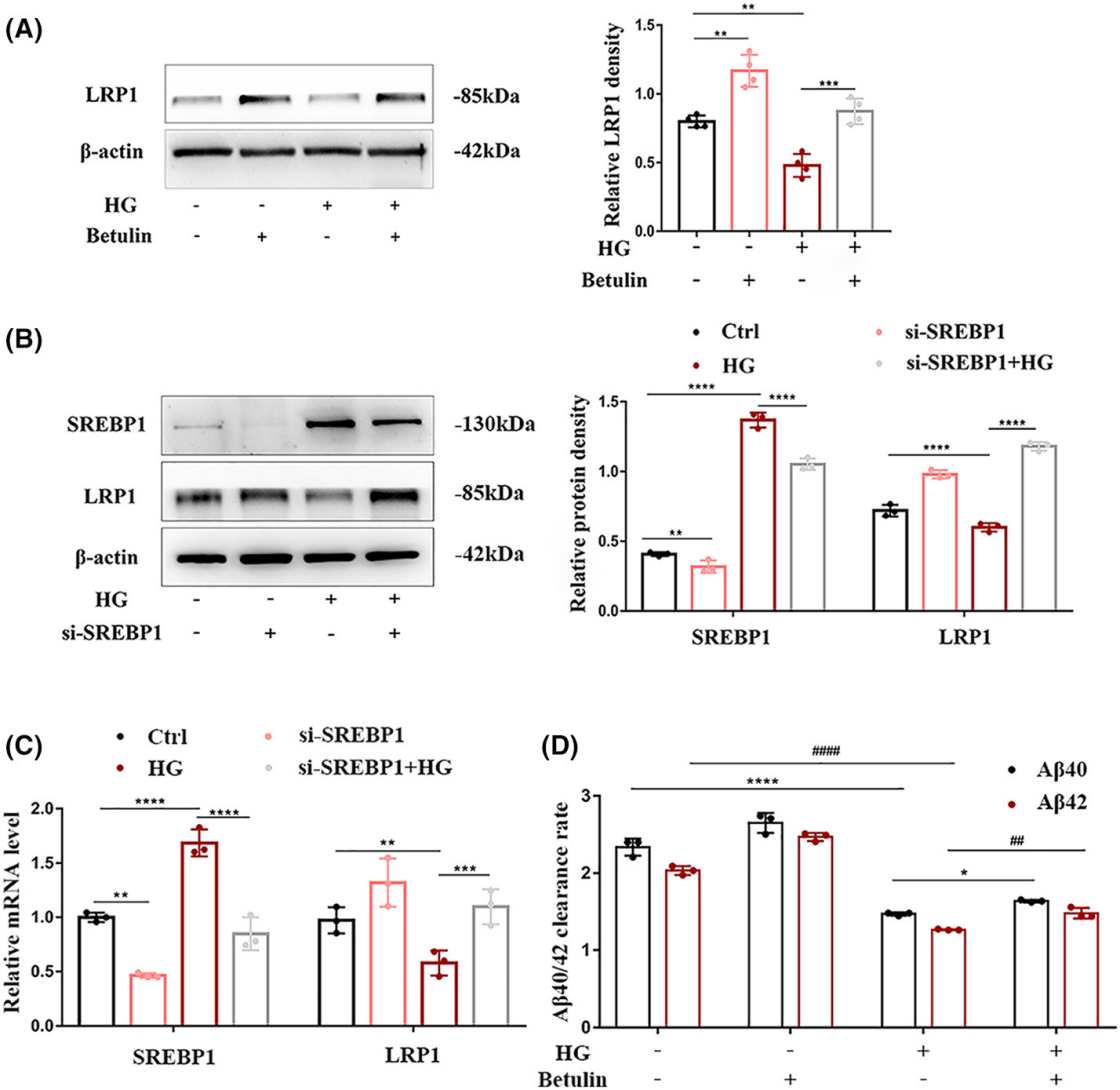
SREBP1 damages Aβ efflux by HBMECs under high glucose by downregulating LRP1. Cultured HBMECs pretreated with or without betulin (50 mmol/L) were stimulated with 50 mM glucose. After 72 h, (A) the protein level of LRP1 was determined by Western blotting, (D) and the efflux rate of Aβ was evaluated in a Transwell in vitro blood–brain barrier model. (B, C) HBMECs were transfected with SREBP1 siRNA (si‐SREBP1) and the corresponding negative control (Ctrl.), followed by 50 mM glucose stimulation for 72 h, and then lysis. Western blotting with SREBP1 and LRP1 antibodies was performed, and relative mRNA expression was detected by Quantitative reverse transcription–polymerase chain reaction (qRT‐PCR). mRNA expression was normalized to β‐actin. Data are shown from at least three independent experiments. *,^#^ *p* < 0.05; **,^##^ *p* < 0.01; ***,^###^ *p* < 0.001 and ****,^####^ *p* < 0.0001.

To determine whether SREBP1 is involved in high glucose‐induced LRP1‐mediated Aβ efflux disorder in the cerebral vascular endothelium, we targeted SREBP1 with inhibitors, silenced genes and measured the mRNA and protein expression levels of SREBP1 and LRP1 in HBMECs stimulated with high glucose by real‐time quantitative PCR and Western blotting. The results showed that the expression of LRP1 decreased under high‐glucose stimulation. When SREBP1 was inhibited, the downregulation of LRP1 expression caused by high glucose was improved (Figure 4A–C). Consistent with our hypothesis, when betulin and SREBP1 siRNA (si‐SREBP1) were used to reduce the levels and activity of SREBP1, the decrease in Aβ efflux induced by high glucose was significantly improved (Figure 4D), which was consistent with the change in LRP1 expression.

### 3.5 mTORC1 regulates the SREBP1/LRP1 pathway in Raptor^fl+^ diabetic mice

**FIGURE 5:**
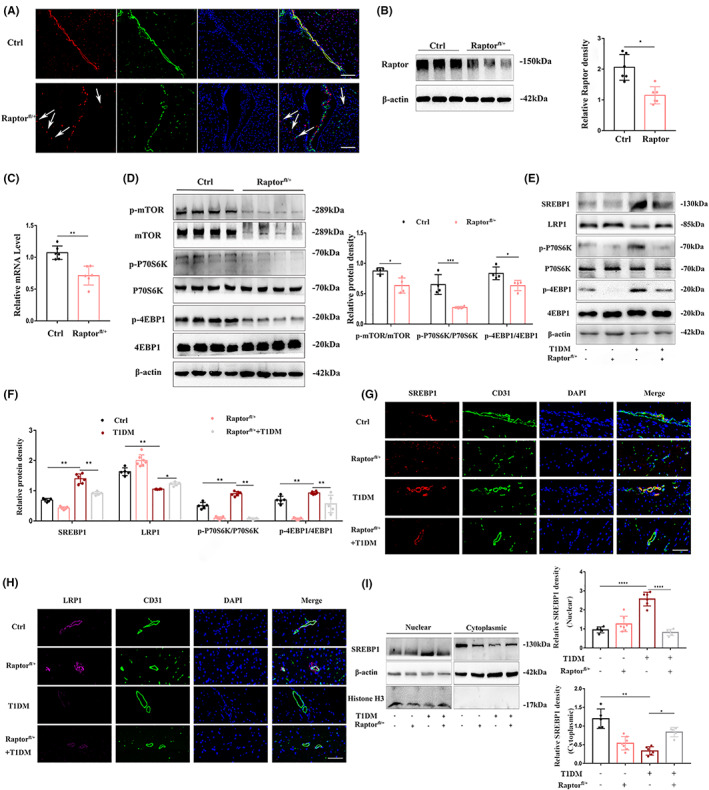
mTORC1 regulates the SREBP1/LRP1 pathway in Raptor^fl+^ diabetic mice. (A) Raptor was knocked down in Raptor^fl/+^ mice. Endothelial markers CD31 and Raptor immunofluorescence double staining in cortical brain slices showed that Raptor was knocked down in the brain endothelium of Raptor^fl/+^ mice, while the expression of Raptor in surrounding tissues (nonendothelial) was not affected (white arrow). Scale bar: 100 μm. Isolation of cerebral vascular endothelial cells, (B) Western blotting was used to detect the protein expression of Raptor protein, and β‐actin was used as the internal control (left). The bar graph shows the relative expression presented as the ratio to β‐actin (right). (C) Raptor mRNA expression was detected by quantitative reverse transcription–polymerase chain reaction (qRT‐PCR). mRNA expression was normalized to β‐actin. The number of mice is six. (D) The protein levels of p‐mTOR, mTOR, p‐P70S6K, P70S6K, p‐4EBP1, and 4EBP1 in the whole cell lysates were detected by Western blotting (left). The bar graph shows the quantification of all target proteins (right). The number of mice is four. (E) Protein lysate of mouse brain microvessels was analyzed by Western blotting with p‐P70S6K, P70S6K, p‐4EBP1, 4EBP1, SREBP1, and LRP1 antibodies. (F) The bar graph shows the quantification normalized to β‐actin. The number of mice in each group was five, seven, six, and five, respectively. (G, H) Immunofluorescence staining of SREBP1 (red) and LRP1 (purple) in brain tissue. Cell nuclei were stained with DAPI (blue). Scale bar: 20 μm. *n* = 3 per group. (I) Cytoplasmic and nuclear extracts of mice brain microvessels were immunoblotted using anti‐SREBP1 (left). Bar graphs show the densitometric analysis of SREBP1 expression in cytoplasmic extracts and nuclear extracts (right). The number of mice in each group was five, seven, six, and five, respectively. Data are showed as mean ± SEM of at least three independent experiments. *p < 0.05, **p < 0.01 and ****p < 0.0001.

To clarify the physiological effect of mTORC1 on diabetic cognitive impairment in cerebral vascular endothelial cells, we established a mouse model. The expression of Raptor in the cerebral vascular endothelial cells of control mice was not affected, but in Raptor^fl/+^ mice, Raptor expression in brain tissue was ablated, as shown by double immunofluorescence staining of CD31 positive regions in brain tissue with the monoclonal antibody against Raptor (Figure 5A). Cleavage protein analysis showed that expression in Raptor^fl/+^ mice was decreased by approximately 50% compared with that in the control group (Figure 5B). Genomic DNA gel electrophoresis and qRT‐PCR analysis confirmed the excision of Raptor (Figure 5C; Figure S2A). In addition, after Raptor, which is an important component of mTORC1, was knocked down, the phosphorylation levels of mTOR and its activation markers in the cerebral vascular endothelium were reduced (Figure 5D). We successfully established a diabetic model in Raptor^fl/+^ mice to verify the regulatory mechanism we observed in vitro (Figure 2B). Consistent with our hypothesis, the levels of p‐P70S6K1 and p‐4EBP1 in the brain microvasculature of diabetic mice with specific gene knockout were significantly lower than those in the diabetes group, and the diabetes‐induced activation and induction of mTORC1 was significantly inhibited (Figure 5E,F). In addition, the expression of SREBP1 decreased and LRP1 increased in Raptor^fl/+^ mice (Figure 5E,F), and compared with diabetic mice, the expression of SREBP1 decreased, and the expression of LRP1 increased in Raptor^fl/+^ diabetic mice. Immunofluorescence double staining of frozen sections of brain tissue showed that in the positive region expressing the vascular endothelial cell marker CD31, the fluorescence intensity of SREBP1 decreased in conditional gene knockout mice, while LRP1 increased (Figure 5G,H). It is satisfying that the nuclear accumulation of SREBP1 in diabetic mice was also observed at the tissue level, while the nuclear accumulation was reversed in Raptor^fl/+^ diabetic mice (Figure 5I). Based on these results, it can be concluded that diabetes induced mTORC1 activation, upregulated the expression of SREBP1, caused its translocation, and then downregulated LRP1.

### 3.6 mTORC1 is essential for Aβ deposition and cognitive impairment in diabetic mice

**FIGURE 6:**
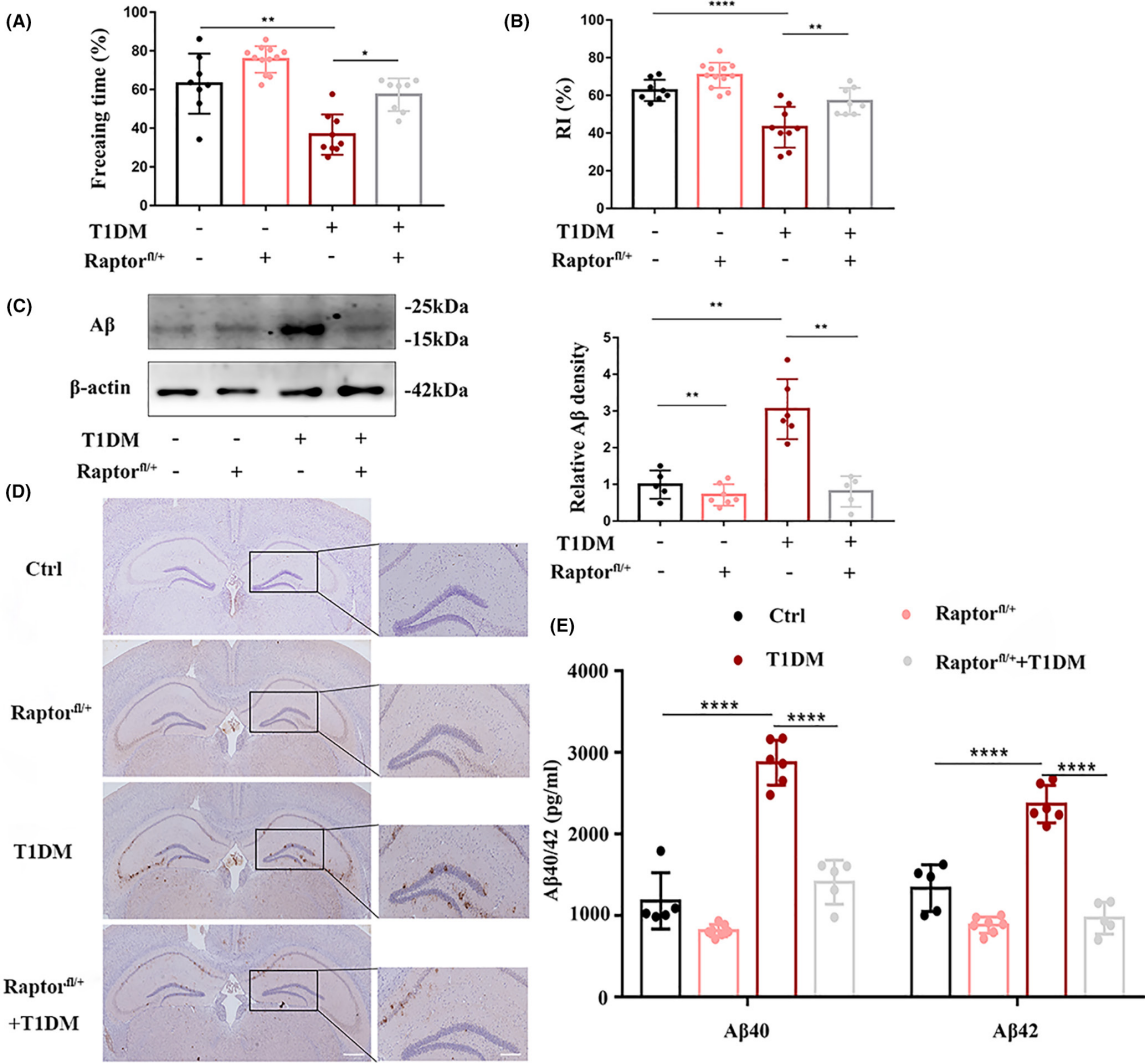
mTORC1 is essential for Aβ deposition and cognitive impairment in diabetic mice. (A) The fear conditioning test and (B) novel object recognition test were used to examine learning and memory functions in mice. The numbers in the Ctrl., Raptor^fl/+^, T1DM, and Raptor^fl/+^+T1DM groups were eight, twelve, nine, and eight, respectively. (C) Protein lysate of mouse brain microvessels was analyzed by Western blotting with anti‐Aβ (left). The bar graph (right) shows the quantification normalized to β‐actin. The number of mice was five, seven, six, and five, respectively. (D) Immunohistochemical staining of Aβ in the hippocampus. Scale bars: 1 mm and 100 μm, respectively. (E) ELISA was used to determine the levels of Aβ in brain homogenates in each group. The number of mice in each group was consistent with (C). Representative results of three repeated experiments are shown. **p < 0.01, ***p < 0.001 and ****p < 0.0001.

Next, we aimed to determine the role of mTORC1 in cerebrovascular endothelial cells in vivo by analyzing the differences in Aβ levels and cognitive function. Compared with that of the control group, the freezing time of diabetic mice decreased by 20% in the fear conditioning test. Similarly, in the novel object recognition test, the number of explorations new objects by diabetic mice decreased significantly, accounting for 18%. This result indicated that diabetes seriously affected the memory function of mice. In Raptor^fl/+^ diabetic mice, the freezing time and the frequency of exploring the new object were significantly increased (Figure 6A,B). Then, we used different methods to verify the effect of mTORC1 resection on Aβ metabolism from various dimensions. Western blotting showed that diabetes led to an increase in Aβ deposition in brain tissue, while after inhibiting mTORC1 in the cerebral vascular endothelium, Aβ deposition decreased significantly (Figure 6C). Immunohistochemical staining of Aβ revealed that diabetes failed to trigger an effective increase in the area and number of Aβ plaques in the hippocampus of Raptor^fl/+^ mice (Figure 6D). ELISA analysis of Aβ40 and Aβ42 in brain homogenate showed that with the removal of mTORC1 from the cerebral vascular endothelium, the deposition of Aβ in the brain caused by diabetes was improved (2900 pg/mL vs. 1200 pg/mL; 2200 pg/mL vs. 900 pg/mL, respectively; Figure 6E). Interestingly, we found that the number of TUNEL‐positive cells in the hippocampus of diabetic mice was much higher than that in the control group, and the number of TUNEL‐positive cells decreased after removal of cerebrovascular endothelial cells (Figure S3A). In addition, HE staining of brain tissue paraffin sections showed that the arrangement of neurons in the hippocampal CA1 region in diabetic mice was loose and scattered, the nucleus in nerve cells was broken, and vacuole‐like changes were observed in the cells. Although some nerve cells were degenerated in Raptor^fl/+^ mice, the arrangement of the cells was regular and the morphology was normal (Figure S3B). These observations showed that mTORC1 in the brain microvascular endothelium was necessary for diabetes‐induced Aβ deposition and memory impairment.

## 4 DISCUSSION

In this study, our results show for the first time that brain microvascular endothelial mTORC1 not only participates in neuronal apoptosis but also downregulates the expression of LRP1 by promoting the nuclear translocation of SREBP1, leading to Aβ deposition and cognitive impairment in the brains of diabetic mice. In cultured HBMECs, inhibiting of mTORC1 activity significantly inhibited the expression and activation of SREBP1, while the change in the expression of LRP1 was reversed, and the efflux rate of Aβ in the simulated blood–brain barrier model was also significantly improved. These results reveal the mechanism by which brain microvascular endothelial mTORC1 mediates Aβ clearance disorders and cognitive impairment in diabetes.

To the best of our knowledge, there have been no or very few studies using endothelial cell‐specific promoter‐controlled Cre recombinase to study the role of cerebrovascular endothelial cells in Aβ clearance. Previous studies have focused on the degradation of Aβ by neurons, astrocytes, microglia, vascular smooth muscle cells, and autolysosomes. However, the current literature has emphasized the contribution of the blood–brain barrier to Aβ clearance,^25^ and cerebrovascular endothelial cells, which are an important part of the blood–brain barrier, should also be considered.^26^ Studies have shown that the mTORC1‐regulated protein Raptor is an mTOR‐binding partner that mediates mTOR signaling to downstream targets. Various signaling molecules activate Raptor phosphorylation, thereby inhibiting mTORC1 activity.^27^, ^28^ It has been reported that the Raptor is a switch that triggers the mTORC1 signaling pathway in diabetes.^29^ Therefore, in this study, Raptor^fl/+^ mice were used to exclude the effects of other brain cells and tissues and to avoid the manipulation of mTORC2 signaling, which is known to occur during long‐term rapamycin treatment^30^ to explore the role of mTORC1 in the efflux of Aβ from cerebrovascular endothelial cells.

In our experiment, the cultured HBMECs showed impaired Aβ efflux after high‐glucose stimulation. Furthermore, the expression of LRP1 was reduced, which was also confirmed by qRT‐PCR and immunofluorescence analysis. Interestingly, high glucose‐induced Aβ efflux damage was corrected by the overexpression of LRP1, which had been demonstrated in our previous data.^31^ These results suggest that high glucose affects the efflux of Aβ through the blood–brain barrier by reducing the expression of LRP1 in the cerebral vascular endothelium. Next, we further explored the regulatory mechanisms of high glucose, Aβ clearance disorder, and cerebral vascular endothelial LRP1. mTOR plays an important role in diabetic vascular complications. In high glucose‐induced umbilical vein endothelial cells, mTOR activation aggravates endothelial cell dysfunction and death, thereby affecting vascular function.^32^ In rat retinal microvascular endothelial cells, it was found that under high‐glucose stimulation, mTOR pathway activation mediated the epithelial–mesenchymal transition and promoted the progression of diabetic nephropathy.^33^ In this study, we examined HBMECs under high‐glucose culture and found that mTOR and its phosphorylation levels, which indicated activation were increased in a concentration‐dependent manner. In addition, rapamycin and gene silencing of Raptor showed that high glucose reduced not only the activation of mTOR in the cerebral vascular endothelium but also reversed the efflux of Aβ. Our study showed for the first time that inhibiting mTORC1 could effectively improve the decrease in LRP1 in cerebral vascular endothelial cells induced by high‐glucose injury and improve Aβ efflux.

It is worth noting that our study also showed that SREBP1 was the downstream effector of mTORC1 on LRP1. SREBP1 is an important transcription factor that mediates insulin and glucose to regulate lipid synthesis.^34^ Experimental evidence has shown that high‐glucose treatment in vitro or hyperglycemia in vivo can induce SREBP1 protein expression and nuclear translocation activation in endothelial cells.^35^ In diabetic mice, activation of SREBP1 causes endothelial cell dysfunction and promotes vascular inflammation and atherosclerosis.^36^ Our results showed that the expression of SREBP1 increased with increasing glucose concentrations in cerebrovascular endothelial cells in a high‐glucose environment, and nuclear translocation occurred. After inhibiting SREBP1 with betulin, the high glucose‐induced decrease in LRP1 and Aβ efflux disorders were improved. More importantly, our study also showed that mTOR activity was directly related to SREBP1 expression and LRP1‐mediated Aβ efflux. Fatty acids and glucose induced the transcription of SREBP1 by activating the mTORC1 signaling pathway under obesity and hyperglycemia.^24^ In this study, after the application of rapamycin, the nuclear accumulation of SREBP1 induced by high glucose was significantly reduced. After gene silencing of Raptor, the expression of SREBP1 under high‐glucose stimulation was inhibited. These data demonstrate that the abnormal expression and activation of SREBP1 in the cerebral vascular endothelium are important mechanisms by which mTORC1 regulates LRP1‐ mediated Aβ efflux disorder.

After in vitro confirmation that mTORC1/SREBP1/LRP1 regulates the Aβ efflux mechanism, we constructed a diabetes model and further verified these effects in vivo. Consistent with in the vitro experiments, we found that cerebrovascular endothelial mTORC1 activation decreased, SREBP1 expression decreased, and the decrease in LRP1 expression was corrected in Raptor^fl/+^ diabetic mice compared with control mice. As expected, the levels of Aβ in the brain also decreased accordingly. In tuberous sclerosis^37^ and vascular dementia,^38^ the mTORC1 signaling pathway is highly correlated with cognitive impairment. Therefore, we explored the effect of inhibiting endothelial mTORC1 on cognitive function in diabetes. Our results showed that the memory function of Raptor^fl/+^ diabetic mice was significantly improved. Overall, brain cerebrovascular endothelial mTORC1 may be a therapeutic target for diabetic cognitive impairment.

However, our research also has certain limitations. First of all, although our Transwell method has been widely used in the study of blood–brain barrier models in vitro and is easy to use and reproducible, there is no way to simulate the complexity of the microenvironment in vivo, such as cell–cell or cell–matrix interactions. Second, we only examined the regulatory mechanism of mTORC1 in male Raptor^fl/+^ diabetic mice. Although the mechanism is not fully elucidated, studies have shown that women may have protective factors against cognitive impairment in diabetes. Therefore, mTORC1, which is a potential therapeutic target, needs to be examined in female mice to assess sex differences. However, at least in male mice, we demonstrated the specific mechanism of mTORC1 in diabetic cognitive impairment.

## 5 CONCLUSIONS

In summary, our results have confirmed that inhibiting mTORC1 in the brain microvascular endothelium ameliorates diabetic Aβ brain deposition and cognitive impairment via the SREBP1/LRP1 signaling pathway. This finding supports mTORC1 as a potential therapeutic target in brain microvascular endothelial cells and lays the foundation for the discovery of a specific molecular mechanism for the early prevention and treatment of diabetic cognitive impairment.

## AUTHOR CONTRIBUTIONS

Conceptualization and Funding Acquisition: Wei Li; Investigation, Methodology, Project Administration: Gege Jiang, Zhenzhen Long, Ping Xue, and Mining Chen; Software and Visualization: Yaoling Wang and Kang Yang; Art and Drawing Instruction: Gege Jiang and Yaofeng Wang; Writing‐Original Draft Preparation: Gege Jiang, Zhenzhen Long; Writing‐Review & editing: Gege Jiang and Zhenzhen Long.

## FUNDING INFORMATION

This study was supported by the National Natural Science Foundation of China to W.L. (Grant Nos. 81570745, 81974113).

## CONFLICT OF INTEREST STATEMENT

None.

## Supporting information


SupinfoS1

## Data Availability

The datasets used and/or analyzed during the current study are available from the corresponding author upon reasonable request.
