# Supplementary material for: Inhibition of mammalian target of rapamycin complex 1 in the brain microvascular endothelium ameliorates diabetic Aβ brain deposition and cognitive impairment via the sterol‐regulatory element‐binding protein 1/lipoprotein receptor‐associated protein 1 signaling pathway
Source: CNS Neurosci Ther. 2023 Mar 8;29(7):1762–75. doi: 10.1111/cns.14133 (PMC10324353; doi:10.1111/cns.14133)

**Supplementary materials**

**Inhibition of mTORC1 in the brain microvascular endothelium ameliorates diabetic Aβ brain deposition and cognitive impairment via the SREBP1/LRP1 signaling pathway**

**Supplementary material 1**

**Reagents and Antibodies**

1640 medium was purchased from ScienCell Research Laboratories (Carlsbad, CA). Rapamycin and Betulin were purchased from Selleck (Houston, USA). PrimeScript RT Master Mix kit and SYBR Premix Ex Taq kit were purchased from Vazyme Biotech Co., Ltd. (Nanjing, China). TAMRA-Aβ40 and 42 were purchased from AnaSpec (California, USA). FITC-inulin was purchased from TdB Labs (Uppsala, Sweden). Aβ40 and 42 ELISA kit were acquired from Bioswamp (Wuhan, China). Primary antibodies against p-4EBP1(cst9451), 4EBP1(cst9644), p-p70S6 Kinase(cst9206), p70S6 Kinase(cst2708), p-mTOR(cst5536), mTOR(cst2983) were obtained from Cell Signaling Technology (Danvers, USA). Primary antibodies against β-Amyloid (Cat#sc-28365) and Raptor (Cat#sc-81537) were purchased from Santa Cruz Biotechnology (Dallas, TX, USA). Primary antibody against LRP1(ab92544) was purchased from Abcam (Cambridge, MA). Histone H3(Cat#AF0863, RRID: AB_2810277), Raptor (Cat#DF7527, RRID: AB_2841026) and SREBP1 (Cat#AF6283, RRID: AB_2835134) were obtained from Affinity Biosciences (Ohio, USA). HRP-conjugated secondary antibodies were obtained from Boster Biological Technology (Wuhan, China).

**Supplementary Table 1**

**Information for primer sequences used in Section 2.7**

| **Name** | **Primer** | **Sequence** |
| --- | --- | --- |
| Human  LRP1 | Forward | 5’-GTGACGGAGACAACGATTGC-3’ |
|  | Reverse | 5’-AAGTGGCATTGGACTCATCTTC-3’ |
| Mouse  LRP1 | Forward | 5’-GACCGATTCAAGTGTGAGAACA-3’ |
|  | Reverse | 5’- TCGCTGTTGCCACAATCATTAT-3’ |
| Human  SREBP1 | Forward | 5’- CTGTGTGACCTGCTTCTTGT-3’ |
|  | Reverse | 5’- CTCATGTAGGAACACCCTCC-3’ |
| Mouse  SREBP1 | Forward | 5’-GCACTGAGGCAAAGCTGAATA-3’ |
|  | Reverse | 5’-CGACACCAGATCCTTCAGAGA -3’ |
| Human  Raptor | Forward | 5’-GACACGGAAGATGTTCGACAAG-3’ |
|  | Reverse | 5’-ATCTGAGAAGCAACGCTCTC-3’ |
| Mouse  Raptor | Forward | 5’-ATCTCTTCCAAAAGCTTTTCCG-3’ |
|  | Reverse | 5’-TACTGGGGTGCAGTTATATGAC-3’ |
| Human  β-actin | Forward | 5’- CCTGGCACCCAGCACAAT-3’ |
|  | Reverse | 5’-GGGCCGGACTCGTCATAC-3’ |
| Mouse  β-actin | Forward | 5’-GTGCTATGTTGCTCTAGACTTCG-3’ |
|  | Reverse | 5’-ATGCCACAGGATTCCATACC-3’ |

Abbreviations: LRP1 low-density lipoprotein receptor-associated protein 1, SREBP1 Sterol-regulatory element binding protein 1, Raptor regulatory associated protein of mTOR.

**Supplementary Figure 1 Transendothelial electrical resistance (TEER) values.** TEER values of the HBMECs determination by the EVOM2 voltmeter under different times. The data are showing the representative results of six repeated experiments.

**
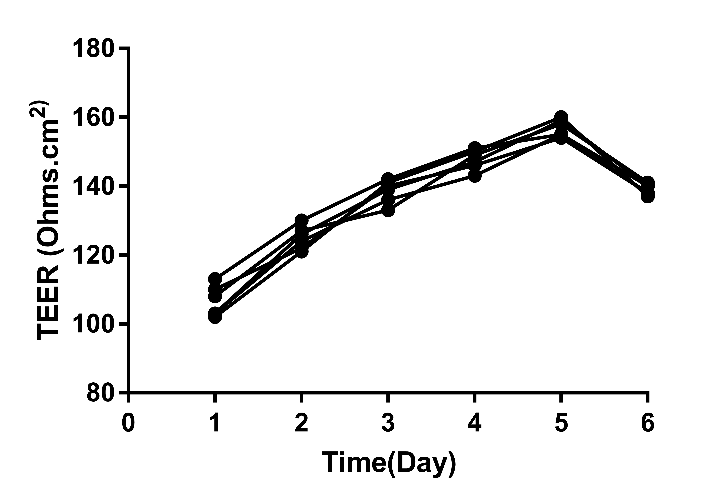
**

**Supplementary Figure 2 Successful establishment of cerebrovascular endothelial Raptor specific knockout mice and diabetic mice models.** (A) Genotyping of DNA Extracts from Tail of Specific Condition Knockout Mice by DNA Gel Electrophoresis­ (**left, Raptor; right, Cre**). (B) Blood glucose of diabetic mice in each group. The number of Ctrl., Raptor^fl/+^, T1DM and Raptor^fl/+^+T1DM group is eight, twelve, nine and eight, respectively. The data are expressed as mean ± SD.


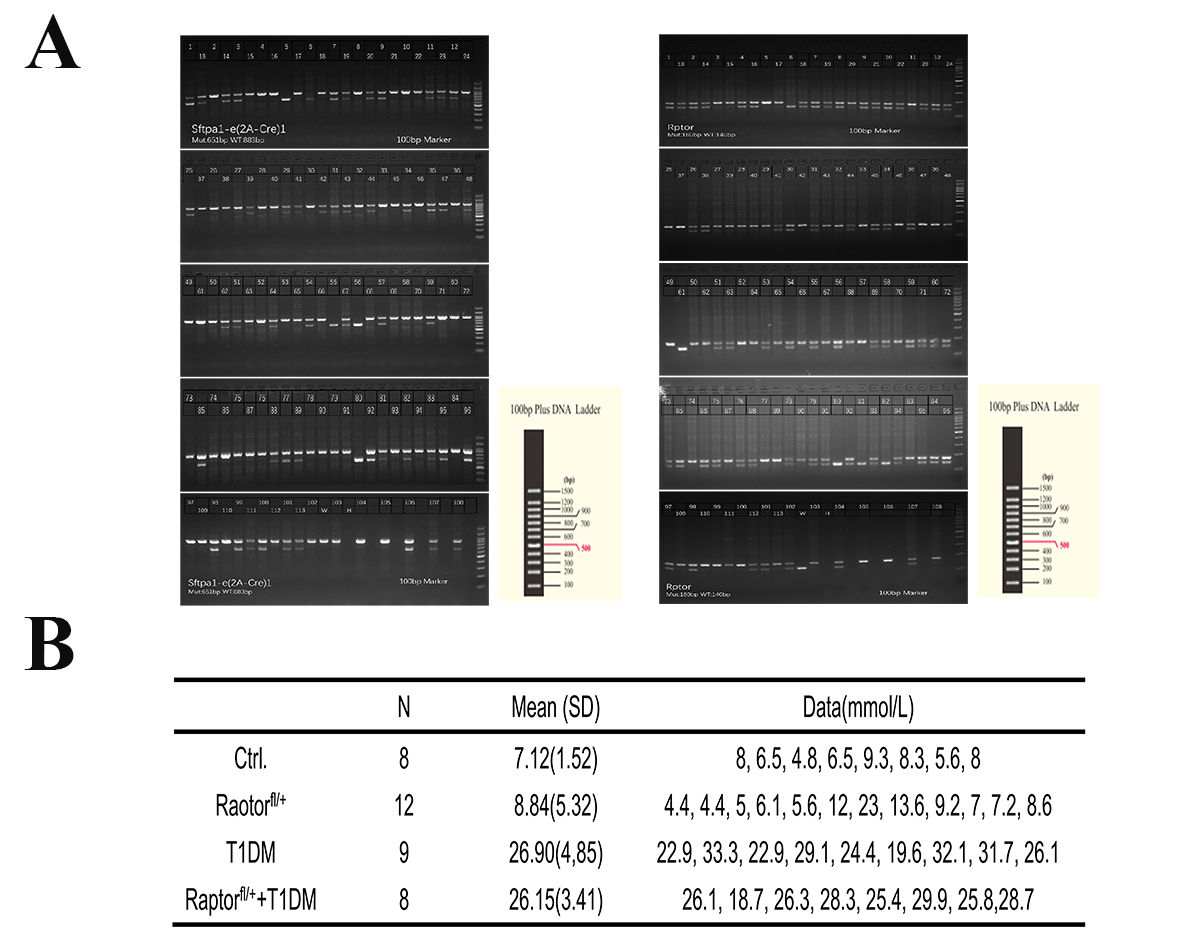


**Supplementary Figure 3 mTORC1 of BMECs is essential for neuronal necrosis and apoptosis in diabetic mice. (A)** Detection of neuronal apoptosis in mouse hippocampus by TUNEL (green). Cell nuclei were stained with DAPI (blue). Scale bar:200μm. (B) HE staining observed the morphology of hippocampal CA1 neurons. Scale bar: 100μm. n=3 per group. The data are showing the representative results of three repeated experiments.


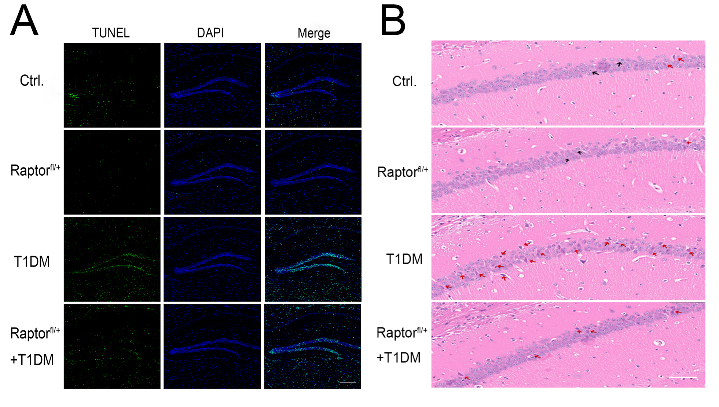

Supplement: Supplementary file 1 — SupinfoS1 [file CNS-29-1762-s001.docx]
